# TRIM72-mediated degradation of the short form of p62/SQSTM1 rheostatically controls selective autophagy in human cells

**DOI:** 10.1186/s40779-022-00392-1

**Published:** 2022-06-22

**Authors:** Cheng-Cheng Wang, Hong Peng, Zi Wang, Jiao Yang, Rong-Gui Hu, Chuan-Yin Li, Wu-Jun Geng

**Affiliations:** 1 Cancer Center, Shanghai Tenth People’s Hospital, School of Medicine, Tongji University, Shanghai, 200040 China; 2 Guizhou University School of Medicine, Guiyang, 550025 China; 3 MOE Key Laboratory of Tropical Disease Control, Centre for Infection and Immunity Study (CIIS), School of Medicine, Sun Yat-Sen University, Shenzhen, 510275 Guangdong China; 4 Institute for Frontier Life and Medical Sciences, Kyoto University, Kyoto, 606-8501 Japan; 5 State Key Laboratory of Molecular Biology, Shanghai Institute of Biochemistry and Cell Biology, Center for Excellence in Molecular Cell Science, Chinese Academy of Sciences, Shanghai, 200031 China; 6 Department of Anesthesiology, Wenzhou Key Laboratory of Perioperative Medicine, the First Affiliated Hospital of Wenzhou Medical University, Wenzhou, 325000 Zhejiang China

**Keywords:** p62S, Alternative splicing, Ubiquitination, TRIM72, Autophagy

Dear Editor,

Autophagy is an evolutionarily conserved catabolic process that involves the sequestration and transport of organelles, macromolecules, or invading microorganisms to lysosomes for degradation [1]. Sequestosome 1 (p62/SQSTM1) was the first protein shown to bind target-associated ubiquitin (Ub) and LC3 conjugated to the phagophore membrane, thus, acting as an important autophagy receptor for ubiquitinated targets [2].

Human *p62/SQSTM1* has alternative 5’donor sites during splicing of immature mRNA, which results in two isoforms of the *p62/SQSTM1* transcripts, *p62L* and *p62S*. In contrast to p62L, differences in the 5’-untranslated region (UTR) and coding sequence (CDS) region between the two transcript variants resulted in a loss of 1–84 amino acids (included within the PB1 domain) in p62S (Fig. 1a, Additional file 1: Fig. S1a). In this study, two pairs of primers were designed to specifically amplify the cDNA fragments of *p62* isoforms in different cell lines (Additional file 1: Table S1). Expression of *p62L* was detected in all 9 cell lines, whereas *p62S* expression was detected in most cell lines, with the exception of human skeletal muscle cells (HSkMC) and adult hepatocytes (Additional file 1: Fig. S1b). No p62S expression was found in various tissues of mice (Additional file 1: Fig. S1c), indicating a human cell-type-specific expression pattern in the biogenesis of p62S. An shRNA specifically targeting *p62S* but not *p62L* was designed (Additional file 1: Table S2), and validated by quantitative PCR and immunoblot analysis (Additional file 1: Fig. S1d), indicating the presence of p62S.

**Fig. 1:**
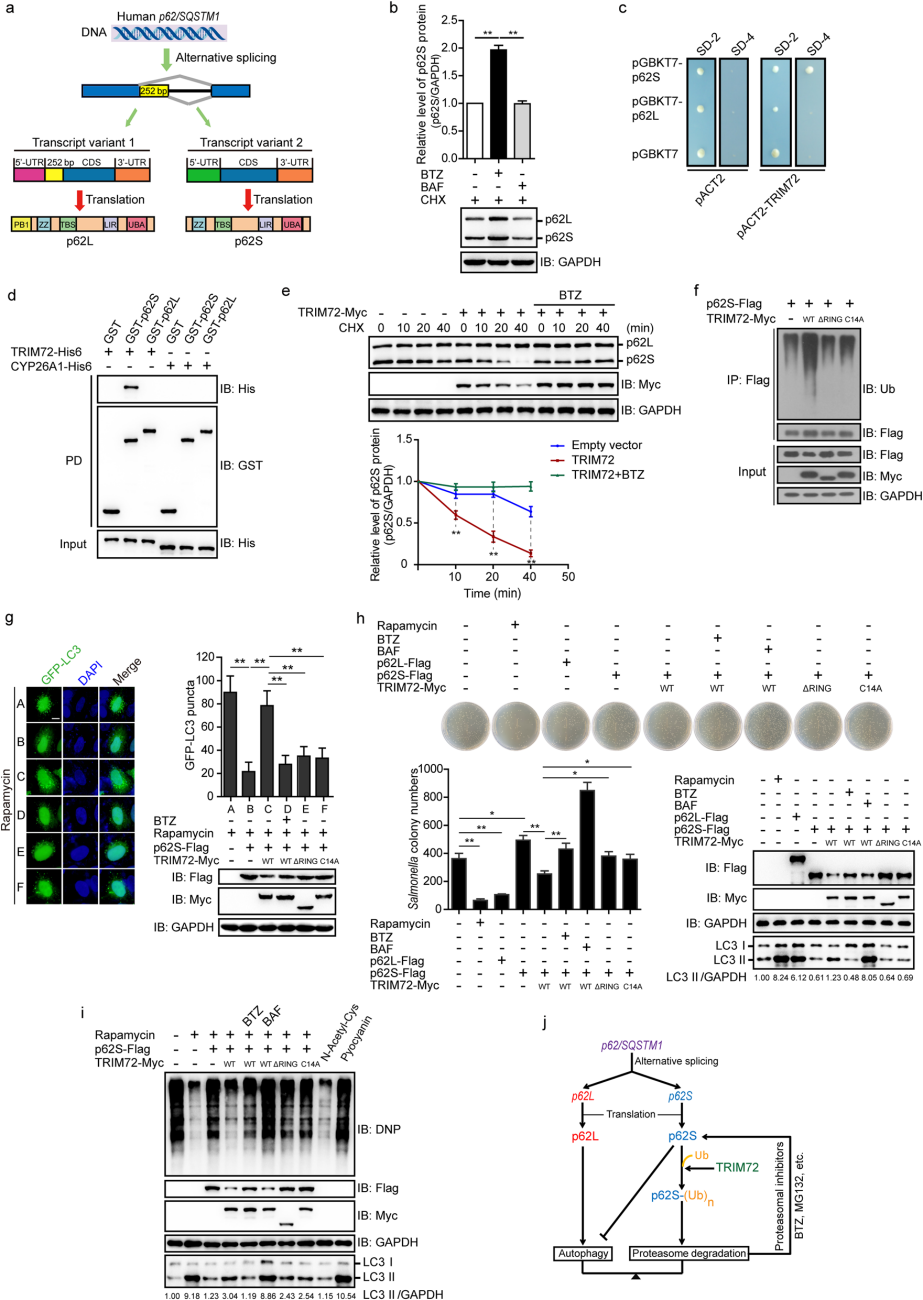
TRIM72-mediated ubiquitin signaling controls the homeostasis of the p62/SQSTM1 short isoform in regulating selective autophagy. **a** Schematic domain structure of p62L and p62S, which were derived from alternative splicing of the human *p62/SQSTM1* gene. **b** Endogenous p62S and p62L were mainly degraded by proteasomes, but not by the autophagy pathway in HeLa cells treated with bortezomib (BTZ, 1 μmol/L) or bafilomycin (BAF, 20 nmol/L), as well as cycloheximide (CHX, 100 μg/ml). **c** Yeast two-hybrid screening identified TRIM72 as an interacting partner for p62S, but not for p62L. SD-2 was deficient in Leu and Trp, and SD-4 was deficient in Ura, His, Leu, and Trp. **d** Recombinant glutathione-S-transferase (GST)-tagged p62S directly interacted with His6-tagged TRIM72 in vitro as detected by a GST pull-down (PD) assay. Recombinant His6-tagged CYP26A1 acted as a negative control. **e** TRIM72 promoted the degradation of endogenous p62S, but not p62L, using the proteasome pathway. HeLa cells were ectopically expressed with empty vector or TRIM72, treated with the indicated compounds during different times, and detected by immunoblot analyses. **f** Wild-type TRIM72 but not the E3 ligase death mutants (TRIM72^ΔRING^ and TRIM72^C14A^) supported the poly-ubiquitylation of p62S. HEK293T cells were ectopically expressed with the indicated plasmids, and then the cell lysates were immunoprecipitated with anti-Flag affinity gels before being subjected to immunoblotting analysis. **g** HeLa cells were co-transfected with the indicated plasmids, treated with 2 μmol/L rapamycin for 12 h, then with or without BTZ for 1 h, and subjected to fluorescent microscopy. Puncta formation by GFP-LC3 were counted and calculated. The protein levels of TRIM72 and p62S were also detected by immunoblot analysis. Scale bar = 10 μm. **h** *Salmonella* infection assay indicated that TRIM72 mediated the proteasomal degradation of p62S, and facilitated the clearing of *Salmonella*. HeLa cells were transfected with the indicated plasmids for 24 h, and then treated with the indicated compounds before *Salmonella* infection for 30 min. The cells were then lysed and one-tenth of the lysate was subjected to plate assays. The *Salmonella* colony numbers were counted and calculated. The protein levels of TRIM72, p62L, p62S, and LC3 were also detected by immunoblot analysis, and the expression of lipidated LC3 (LC3 II) was quantitated after normalization of control values as 1.00. **i** TRIM72-mediated proteasomal degradation of p62S facilitated carbonylated protein degradation. HeLa cells were transfected with the indicated plasmids for 24 h, and then treated with the indicated compounds before being subjected to the detection of protein oxidation. Pyocyanin was used as a positive control, and N-acetyl-l-cysteine was used as a negative control. Carbonylated proteins were visualized after derivatization with 2,4-dinitrophenylhydrazine (DNPH), followed by immunoblotting with anti-DNP. The levels of lipidated LC3 (LC3 II) were quantitated after normalization of the control as 1.00. **j** A model showing how TRIM72-mediated proteasomal degradation of p62S rheostatically regulated selective cellular autophagy. Data are presented as the mean ± SD, and analyzed with one-way analysis of variance using the Bonferroni post-hoc test or the two-tailed unpaired *t* test. *P < 0.05, **P < 0.01, three independent experiments. CDS coding sequence, CHX cycloheximide, Myc an epitope tag for protein tagged, TRIM72 tripartite motif-containing 72, Ub ubiquitin, UTR Untranslated region

p62S and p62L protein levels were upregulated when treated with the proteasome inhibitor bortezomib (BTZ), but not with the autophagy inhibitor bafilomycin (BAF, Fig. 1b; Additional file 1: Fig. S2a, b), indicating that p62S and p62L were mainly degraded by the proteasome pathway rather than the autophagy pathway.

Using the yeast two-hybrid screening system, tripartite motif-containing 72 (TRIM72) was identified as interacting with p62S but not with p62L (Fig. 1c). The interaction of TRIM72 and p62S was subsequently verified by glutathione-S-transferase (GST)-pull-down (Fig. 1d) and co-immunoprecipitation assays (Additional file 1: Fig. S3a). TRIM72 promoted the degradation of p62S in a dose-dependent manner (Additional file 1: Fig. S3b), and ectopic expression of TRIM72 decreased p62S protein level and increased lipidated LC3 (LC3 II) level, while TRIM72 knockdown increased p62S level and decreased LC3 II level (Additional file 1: Fig. S3c). Pulse-chase experiments revealed that p62S, but not p62L protein, was reduced upon TRIM72 overexpression, indicating a specific effect of TRIM72 on p62S proteasomal degradation (Fig. 1e). Furthermore, wild-type TRIM72, but not an E3 ligase activity death mutant (TRIM72^ΔRING^ or TRIM72^C14A^), supported the ubiquitination of p62S (Fig. 1f). Together, these results suggest that TRIM72 is a p62S-specific E3 ligase.

Regarding the physiological function of the TRIM72-mediated degradation of p62S protein, PB1 lacking p62S bound to the poly-ubiquitinated cargo and was capable of binding to LC3 as efficiently as p62L (Additional file 1: Fig. S4a, b). Upon treatment with rapamycin, p62S reduced GFP-LC3 puncta formation in HeLa cells, indicating that p62S suppressed rapamycin-induced autophagy, which was reversed by wild-type TRIM72 but not its mutants (TRIM72^ΔRING^ and TRIM72^C14A^, Fig. 1g). In addition, TRIM72 regulated autophagy, mainly through p62S, but not through p62L, as shown in p62^−/−^ MEF cells (Additional file 1: Fig. S4c).

Because invading microorganisms are mainly degraded through autophagy, a *Salmonella* infection assay was used to measure cellular autophagic activities [2]. Introduction of rapamycin and p62L into HeLa cells reduced the number of *Salmonella* infection by increasing autophagy (LC3 II), while introduction of p62S increased the number of infected *Salmonella* by reducing autophagy. When p62S was down-regulated by wild-type TRIM72, the number of infected *Salmonella* were significantly reduced due to increased autophagy (Fig. 1h).

Another function of cellular autophagy is to remove proteins from oxidative damaged (carbonylation) [3]. The degradation of carbonylated proteins in HeLa cells after rapamycin treatment was accelerated by increasing autophagy (LC3 II). This process was reduced in the presence of p62S, and the introduction of wild-type TRIM72 almost abolished the inhibitory effect of p62S, which was abolished by BTZ (Fig. 1i).

Taken together, our results revealed a previously unexplored mechanism involving alternative splicing of *p62/SQSTM1* mRNA with TRIM72-mediated Ub signaling, to rheostatically control the cellular autophagic flux (Fig. 1j). Further exploitation of this mechanism offers new opportunities to develop therapeutic interventions for these related pathophysiological processes.

## Supplementary Information

**Additional file 1: Materials and Methods. Fig. S1** Human p62 has a shorter isoform p62S lacking the PB1 domain at the N-terminus. **Fig. S2** Human p62S is mainly degraded though the proteasome pathway. **Fig. S3** Human E3 ligase TRIM72 mediates the ubiquitination and degradation of p62S-regulated cellular autophagy. **Fig. S4** Human p62S antagonizes the autophagy receptor function of p62L. **Table S1** Sequences of the primers used in RT-PCR and qPCR. **Table S2** Sequences of the shRNAs for p62S and TRIM72.

## Data Availability

All the data and materials generated or analyzed in this study are available for other researchers after the manuscript is published.

## Abbreviations

BTZ: Bortezomib
BAF: Bafilomycin
CDS: Coding sequence
GST: Glutathione-S-transferase
HSkMC: Human skeletal muscle cell
LC3: Microtubule-associated protein 1A/1B-light chain 3
SQSTM1/p62: Sequestosome 1
TRIM72: Tripartite motif-containing 72
Ub: Ubiquitin
UTR: Untranslated region

## References

[1] Klionsky DJ, Abdel-Aziz AK, Abdelfatah S, Abdellatif M, Abdoli A, Abel S (2021). Guidelines for the use and interpretation of assays for monitoring autophagy (4th edition). Autophagy.

[2] Peng H, Yang J, Li G, You Q, Han W, Li T (2017). Ubiquitylation of p62/sequestosome1 activates its autophagy receptor function and controls selective autophagy upon ubiquitin stress. Cell Res.

[3] Liu Z, Chen P, Gao H, Gu Y, Yang J, Peng H (2014). Ubiquitylation of autophagy receptor Optineurin by HACE1 activates selective autophagy for tumor suppression. Cancer Cell.

