# Supplementary material for: TRIM72-mediated degradation of the short form of p62/SQSTM1 rheostatically controls selective autophagy in human cells
Source: Mil Med Res. 2022 Jun 22;9:35. doi: 10.1186/s40779-022-00392-1 (PMC9215040; doi:10.1186/s40779-022-00392-1)
Supplement: Supplementary file 1 — Additional file 1: Materials and Methods. Fig. S1 Human p62 has a shorter isoform p62S lacking the PB1 domain at the N-terminus. Fig. S2 Human p62S is mainly degraded though the proteasome pathway. Fig. S3 Human E3 ligase TRIM72 mediates the ubiquitination and degradation of p62S-regulated cellular autophagy. Fig. S4 Human p62S antagonizes the autophagy receptor function of p62L. Table S1 Sequences of the primers used in RT-PCR and qPCR. Table S2 Sequences of the shRNAs for p62S and TRIM72. [file 40779_2022_392_MOESM1_ESM.pdf]

Materials and Methods

Cell culture and transfection

Human cell lines, HEK293T, HeLa, SHSY5Y, H1299, HCT116, SMMC-7721, HepG2, HSkMC, Hepatocyte, and $p62^{-/-}$ MEF cells, were cultured in Dulbecco's Modified Eagle's Medium supplemented with 10% fetal bovine serum, 100 U/ml penicillin, and 100 mg/ml streptomycin (all from Gibco, Gaithersburg, MD, USA) in a 37°C humidified atmosphere of 5% CO_2. Plasmids used in this study were transfected into HeLa cells using Lipofectamine 2000 (Life Technologies, Carlsbad, CA, USA) according to the manufacturer's instructions.

Reverse transcription PCR (RT-PCR) and quantitative PCR (qPCR)

Total RNA was extracted from HeLa cells using an RNAsimple total RNA kit (Tiangen, Beijing, China) according to the manufacturer's instructions. The cDNA was synthesized using a ReverTra Ace qPCR RT Master Mix (Toyobo, Osaka, Japan). RT-PCR were performed using the 2× Taq MasterMix (Bioer, Hangzhou, China) to assess the abundances of *p62S* and *p62L* mRNAs in different cell lines using specific primers (**Table S1**). *GAPDH* was used as an internal control. The qPCR was conducted using an ABI 7500 Fast Real-Time PCR system (Applied Biosystems, South San Francisco, CA, USA) to assess the relative abundances of *p62S* and *p62L* mRNAs using specific primers (those used in RT-PCR) with staining using SYBR Green (Selleck Chemicals, Shanghai, China). The relative abundances of *p62S* and *p62L* were normalized to that of the *GAPDH* mRNAs, using the $2^{-\Delta\Delta Ct}$ method[1], and data were obtained from three independent experiments.

Plasmid construction

The shRNAs for p62S and TRIM72 were synthesized as oligonucleotides (Biosune, Shanghai, China), annealed, and inserted into the pLKO.1 vector that was digested with EcoRI and AgeI (NEB, Ipswich, MA, USA), to obtain the specific sequences for shRNAs shown in **Table S2**. The plasmids containing p62S, p62L, and TRIM72 were amplified from cDNA human HEK293T or HeLa cells, and inserted into the pCADNA3.0, pET22B, pACT2, pGBKT7, or pGEX4T-1 vector. Compared with p62L, p62S lacked 84 amino acids at the N-terminus (1 − 84 amino acids). The plasmids of TRIM72 (ΔRING), TRIM72 (C14A), and p62L (ΔPB1) were generated using a QuikChange Site-Directed Mutagenesis Kit (Stratagene, San Diego, CA, USA). Plasmids expressing ubiquitin (pRK5-HA-UB) were kindly provided by Professor Ronggui Hu (Chinese Academy of Sciences, Shanghai, China).

Yeast two-hybrid screening

The p62S open reading frame was cloned into pGBKT7, generating the bait plasmid, pGBKT7-

P62S, which contained the in-frame fusion of the GAL4 DNA binding domain. Yeast two-hybrid (Y2H) screening was performed by transforming a yeast strain (Mav203 strain) that contained the bait vector, with the pACT2 prey vectors for the human E3 cDNA expression library. Yeast transformants were first grown on a plate containing SD-2 (deficient in Leu and Trp) for selection of yeast cells containing both bait and prey vectors, and then transferred to SD-4 (deficient in Leu, Trp, His, and Ura) plates to screen for E3 ligase proteins that potentially interacted with human p62S. The interaction was confirmed by transforming yeast Mav203 cells with the indicated bait and prey vectors, and allowing the transformants to grow on the SD-2 or SD-4 agar plates for approximately 3 days at 30°C. Images of the colonies on both plates were recorded.

Co-immunoprecipitation (Co-IP), immunoprecipitation (IP) and immunoblotting (IB)

For Co-IP, HeLa cells transfected with plasmids were lysed in 800 µl Co-IP buffer (50 mmol/L Tris-HCl, 150 mmol/L NaCl, 5 mmol/L EDTA, and 1% NP-40, pH 7.4) supplemented with a protease inhibitor cocktail (Roche, Indianapolis, IN, USA). Cell lysates were centrifuged at 4°C using 13,000 × *g* for 10 min, incubated with anti-Flag immunomagnetic beads (L-1011; Biolinkedin Biotech, Beijing, China) overnight at 4°C, and washed three times with Co-IP buffer. For immunoprecipitation, HeLa cells transfected with plasmids were lysed in 1 ml of IP buffer (50 mmol/L Tris-HCl, 150 mmol/L NaCl, 5 mmol/L EDTA, 0.1% SDS, and 1% NP-40, pH 7.5) supplemented with a protease inhibitor cocktail, and the cell lysates were centrifuged at 4˚C using 15,000 × *g* for 10 min, incubated with anti-Flag affinity gels (L-1013, Biolinkedin Biotech) overnight at 4°C, then washed three times with IP buffer. The immunoprecipitates from Co-IP and IP were denatured at 100°C for 10 min in 2× SDS-PAGE loading buffer. The inputs, immunoprecipitates, and other cell lysates were subjected to SDS-PAGE, and then transferred to a polyvinylidene difluoride (PVDF) membrane (Bio-Rad, Hercules, CA, USA). The membranes were blocked with 10% nonfat milk at room temperature for 1 h, then incubated with the appropriate antibodies against p62 (1:800, GP62C; Progen, Heidelberg, Germany), GAPDH (1:5000, 60004-1-Ig; ProteinTech, Wuhan, China), Myc tag (1:2000, 16286-1-AP; ProteinTech), FLAG (1:5000, 20543-1-AP; ProteinTech), ubiquitin (1:500, sc-47721; Santa Cruz Biotechnology, Santa Cruz, CA, USA), HA (1:2000, SAB3500908; Sigma-Aldrich, St. Louis, MO, USA), TRIM72 (1:500, 22151-1-AP; ProteinTech), or LC3 (1:1000, L7543; Sigma-Aldrich) overnight at 4°C, washed three times with TBST (50 mmol/L Tris-HCl, 150 mmol/L NaCl, and 0.1% Tween-20, pH 7.4), then incubated with horseradich peroxidase-conjugated secondary antibodies [goat anti-mouse IgG (H + L), SA00001-1, 1:5000 dilution; goat anti-rabbit IgG (H + L), SA00001-2, 1:5000 dilution; ProteinTech] at room temperature for 1 h, washed three times with TBST, and the signals visualized by enhanced chemiluminescence (180-5001; Tanon Science and Technology, Shanghai, China) and detected by

exposure to X-ray film, or detected by a 5200 Imaging System (Tanon). The density of bands was calculated using ImageJ software, version 1.8.0 (National Institutes of Health, Bethesda, MD, USA) when needed.

Expression and purification of recombinant proteins

The pGEX4T-1-GST-p62S, pGEX4T-1-GST-p62L, pET22b-TRIM72-His6, pET22b-LC3-His6, and pET22b-CYP26A1-His6 plasmids were expressed in BL21 *Escherichia coli*, then single cells were picked, cultured in 37ºC in 2 ml LB medium (10 g/L Tryptone, 10 g/L yeast extract, and 10 g/L NaCl) with respective resistance overnight, then the bacteria were inoculated into 500 ml LB medium and cultured for 6 h at 37°C, followed by incubation with isopropyl-β-d-thiogalactopyranoside (Sangon, Shanghai, China) induction at 16°C overnight. The next day, bacterial cells were centrifuged and lysated in phosphate-buffered saline (PBS), incubated with glutathione or Ni^{2+}TA beads (GE Healthcare, Indianapolis, IN, USA) to enrich the respective proteins, followed by elution with 50 mmol/L reduced L-glutathione or with 1 mol/L imidazole dissolved in PBS. The eluted products were dialyzed in PBS supplemented with 15% glycerol prior to being aliquoted and preserved at -80°C.

GST pull-down assay

Purified GST-p62S (50 μg), GST-p62L (50 μg), TRIM72-His6 (50 μg), LC3-His6 (50 μg), or CYP26A1-His6 (50 μg) and Glutathione Sepharose 4B (Millipore, Burlington, MA, USA) were incubated at 4°C overnight in 1 ml pull-down buffer (20 mmol/L Tris-Cl, 100 mmol/L NaCl, 5 mmol/L $MgCl_2$, 1 mmol/L EDTA, 1 mmol/L DTT, 0.5% NP-40, and 20 μg/ml bovine serum albumin, pH 7.6). The beads were centrifuged with 2000× *g* at 4°C, and washed five times with pull-down buffer. Subsequently, the recovered beads were denatured at 100°C for 10 min in 2× SDS-PAGE loading buffer and subjected to immunoblotting analysis.

Fluorescence microscopy analysis

HeLa cells were transfected with plasmids containing GFP-LC3, p62S, TRIM72, or its mutants, treated with Rapamycin (2 μmol/L) for 12 h with or without bortezomib (BTZ, 1 μmol/L) for 1 h, fixed with 4% paraformaldehyde for 20 min, and the cell nucleus was counterstained with 4,6-diamidino-2-phenylindole. Fluorescence microscopy was conducted using a BX51 microscope (Olympus, Tokyo, Japan). Puncta formation by GFP-LC3 was quantitated using 10 cells assessed from 10 fields, and GFP-LC3 puncta were counted and calculated.

***Salmonella* infection assay**

An overnight culture of *Salmonella* strain SL1344 was diluted 1:25 and bacteria were grown at 37°C until reaching OD_{600} at an absorbance of 1.0 − 1.2. HeLa cells were transfected with plasmids

containing p62S, p62L, TRIM72, or its mutants for 24 h, treated with or without Rapamycin (2 μmol/L), BTZ (1 μmol/L), or BAF (20 nmol/L) for 1 h, and washed twice with PBS. The infection was conducted in antibiotic-free medium at a multiplicity of infection of 100. *Salmonella* were allowed to invade cells for 30 min, and then washed three times with PBS. The cells were then lysed and subjected to plate assays, and the *Salmonella* colony numbers were counted and calculated. The experiment was repeated three times.

Detection of cellular oxidatively damaged cellular proteins

HeLa cells were transfected with plasmids containing p62S, TRIM72, or its mutants for 24 h, and treated with or without Rapamycin (2 μmol/L) for 12 h, and BTZ (1 μmol/L), or BAF (20 nmol/L) for 1 h. Protein oxidation was determined using the OxyBlot Protein Oxidation Detection Kit (Millipore) according to the manufacturer's instruction as previously described [2,3]. Briefly, cell lysates were incubated for 20 min with 12% SDS, derivatized with DNP, stopped by Oxyblot Neutralization solution, and 3 μg of total proteins for each sample were resolved in SDS-PAGE, followed by transfer to nitrocellulose membranes. The membranes were blocked with the blocking buffer for 45 min at room temperature before incubation with antibodies against DNP (1:2000, D9656; Sigma-Aldrich) for 2 h, and subsequently with goat anti-rabbit horseradish peroxidase-coupled secondary antibodies (1:5000, ProteinTech) for 1 h at room temperature. Finally, the signals were visualized by enhanced chemiluminescence detected using a 5200 Imaging System (Tanon).

Statistical analysis

Data were analyzed using a two-tailed unpaired *t*-test or one-way analysis of variance with a Bonferroni post-hoc test using Prism 5 software (GraphPad Software, San Diego, CA USA). $^{*}P < 0.05$ was considered to be significantly different.

References

1. Xu X, Tao Y, Gao X, Zhang L, Li X, Zou W, et al. A CRISPR-based approach for targeted DNA demethylation. Cell Discov. 2016;2:16009.

2. Peng H, Yang J, Li G, You Q, Han W, Li T, et al. Ubiquitylation of p62/sequestosome1 activates its autophagy receptor function and controls selective autophagy upon ubiquitin stress. Cell Res. 2017;27(5):657-74.

3. Liu Z, Chen P, Gao H, Gu Y, Yang J, Peng H, et al. Ubiquitylation of autophagy receptor Optineurin by HACE1 activates selective autophagy for tumor suppression. Cancer Cell. 2014;26(1):106-20.

a

```
p62L_Human     1  MASLTVKAYLLGKEDAAREIRRFSFCCSPEPEAEAEAAAGPGPCERLLSRVAALFPALRP
p62S_Human     1  ------------------------------------------------------------

p62L_Human    61  GGFQAHYRDEDGDLVAFSSDEELTMAMSYVKDDIFRIYIKEKKECRRDHRPPCAQEAPRN
p62S_Human     1  ------------------------MAMSYVKDDIFRIYIKEKKECRRDHRPPCAQEAPRN
                                          ************************************

p62L_Human   121  MVHPNVICDGCNGPVVGTRYKCSVCPDYDLCSVCEGKGLHRGHTKLAFPSPFGHLSEGFS
p62S_Human    37  MVHPNVICDGCNGPVVGTRYKCSVCPDYDLCSVCEGKGLHRGHTKLAFPSPFGHLSEGFS
                  ************************************************************

p62L_Human   181  HSRWLRKVKHGHFGWPGWEMGPPGNWSPRPPRAGEARPGPTAESASGPSEDPSVNFLKNV
p62S_Human    97  HSRWLRKVKHGHFGWPGWEMGPPGNWSPRPPRAGEARPGPTAESASGPSEDPSVNFLKNV
                  ************************************************************

p62L_Human   241  GESVAAALSPLGIEVDIDVEHGGKRSRLTPVSPESSSTEEKSSSQPSSCCSDPSKPGGNV
p62S_Human   157  GESVAAALSPLGIEVDIDVEHGGKRSRLTPVSPESSSTEEKSSSQPSSCCSDPSKPGGNV
                  ************************************************************

p62L_Human   301  EGATQSLAEQMRKIALESEGRPEEQMESDNCSGGDDDWTHLSSKEVDPSTGELQSLQMPE
p62S_Human   217  EGATQSLAEQMRKIALESEGRPEEQMESDNCSGGDDDWTHLSSKEVDPSTGELQSLQMPE
                  ************************************************************

p62L_Human   361  SEGPSSLDPSQEGPTGLKEAALYPHLPPEADPRLIESLSQMLSMGFSDEGGWLTRLLQTK
p62S_Human   277  SEGPSSLDPSQEGPTGLKEAALYPHLPPEADPRLIESLSQMLSMGFSDEGGWLTRLLQTK
                  ************************************************************

p62L_Human   421  NYDIGAALDTIQYSKHPPPL
p62S_Human   337  NYDIGAALDTIQYSKHPPPL
                  ********************
```

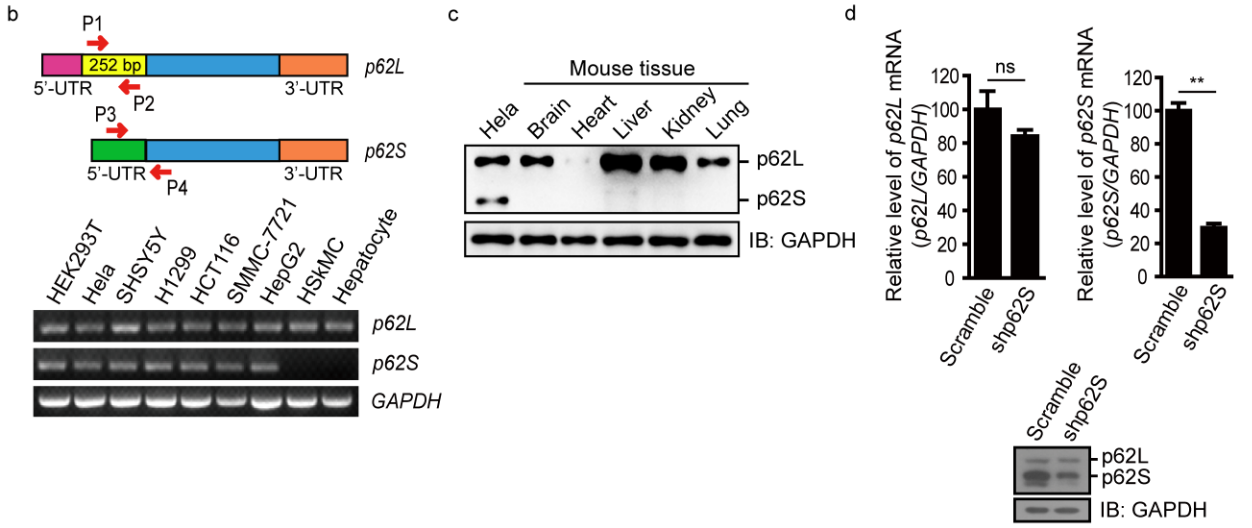

Fig. S1 Human p62 has a shorter isoform p62S lacking the PB1 domain at the N-terminus. **a** Alignment of human p62L with p62S amino acid sequences. Compared with p62L, p62S lacks 84 amino acids at the N-terminus (1 − 84 amino acids). **b** mRNA expression levels of *p62L* and *p62S* in 9 human cells were detected by RT-PCR, which showed that *p62L* was expressed in all 9 different types of cells, and transcripts of *p62S* were not detectable in human skeletal muscle cells (HSkMC) and hepatocytes. Glyceraldehyde 3-phosphate dehydrogenase (GAPDH) was used as an internal control. Primers P1 and P2 were used for the amplification of *p62L* cDNA, and primers P3 and P4 were used for the amplification of *p62S* cDNA. **c** Protein levels of p62L and p62S were detected in human HeLa cells and various mouse tissues by immunoblotting analysis, and no p62S expression was found. **d** shRNAs were designed to only target *p62S* but not *p62L,* to confirm the expression of p62S. Total mRNA extracted from HeLa cells stably transfected with *p62S* scramble or shRNA was used to measure the amount of *p62L* and *p62S* transcripts relative to *GAPDH* by quantitative PCR, and cell lysates were also subjected to immunoblot analysis with the indicated antibodies. Data are presented as the mean ± SD, and analyzed using the two-tailed unpaired *t*-test. $^{**}P < 0.01$, three independent experiments. ns non-significant

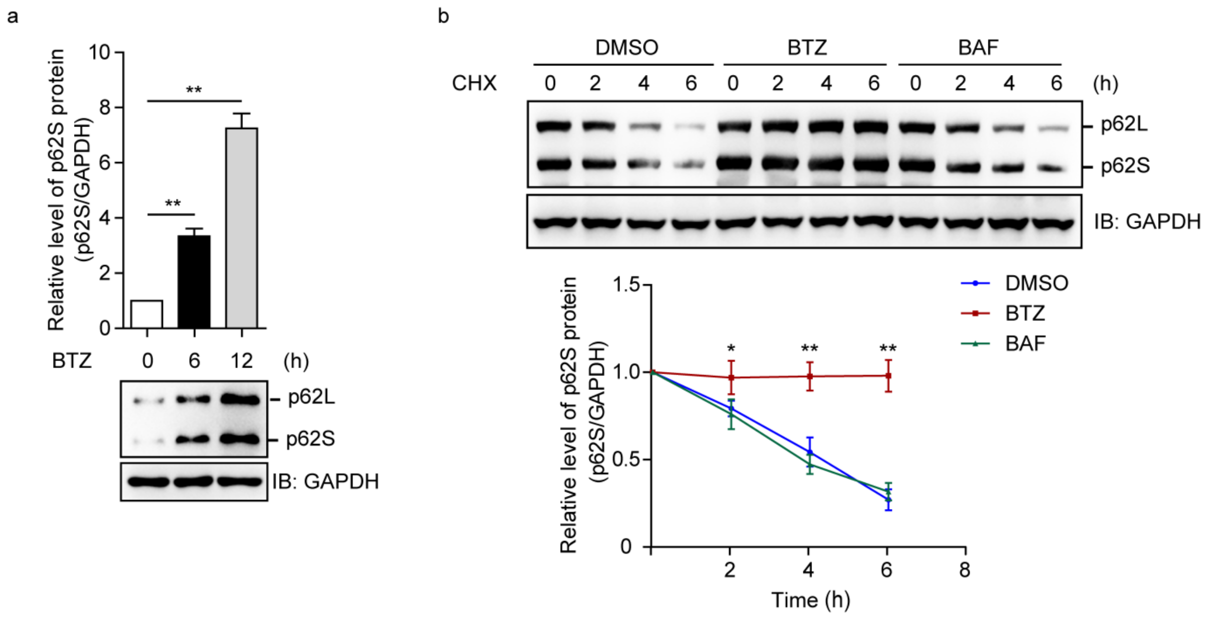

Fig. S2 Human p62S is mainly degraded though the proteasome pathway. **a** Endogenous protein levels of p62L and p62S were both increased when HeLa cells were treated with the proteasome inhibitor, bortezomib (BTZ, 1 μmol/L) for 6 or 12 h, as detected by immunoblot analyses. Data are presented as the mean ± SD, using one-way analysis of variance with the Bonferroni post-hoc test. $^{**}P < 0.01$, three independent experiments. **b** HeLa cells were treated with dimethyl sulfoxide (DMSO), BTZ (1 μmol/L) or bafilomycin (BAF, 20 nmol/L), as well as cycloheximide (CHX, 100 μg/ml), for different times (0, 2, 4, or 6 h) before immunoblotting with the indicated antibodies. BTZ, but not the autophagy inhibitor BAF, inhibited the degradation of p62S. Data are presented as the mean ± SD and analyzed using the two-tailed unpaired *t*-test. $^{*}P < 0.05$, $^{**}P < 0.01$, BTZ groups compared with DMSO groups, three independent experiments. BTZ and BAF were dissolved in DMSO

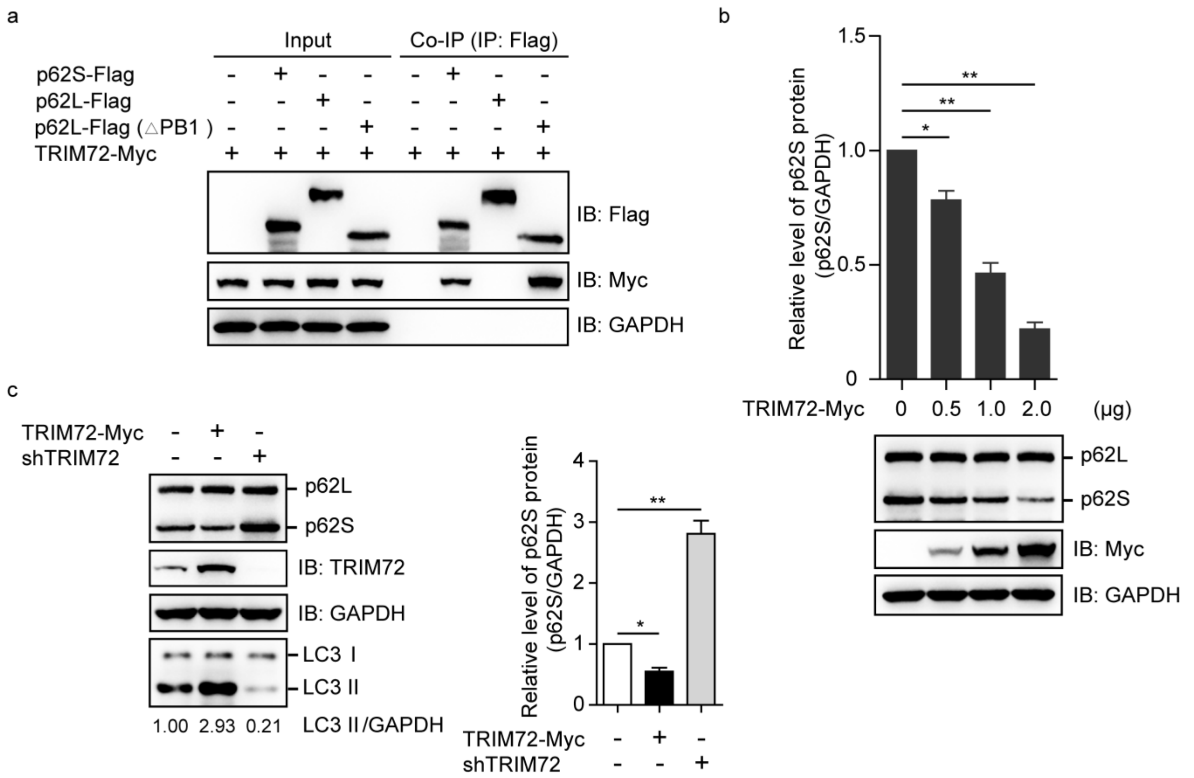

Fig. S3 Human E3 ligase TRIM72 mediates the ubiquitination and degradation of p62S-regulated cellular autophagy. **a** TRIM72 formed a complex with p62S and p62L (ΔPB1) but not with p62L in HEK293T cells, as detected by a co-immunoprecipitation (Co-IP) assay performed using anti-Flag immunomagnetic beads, followed by immunoblotting with anti-Myc and anti-Flag antibodies. **b** TRIM72 promoted the degradation of p62S in a dose-dependent manner. HeLa cells were transiently transfected with different amounts of TRIM72 or empty vectors for 24 h before immunoblotting using the indicated antibodies. **c** TRIM72, but not p62L, regulated endogenous p62S protein levels. HeLa cells were transiently transfected with TRIM72-Myc or shRNA for TRIM72, and subjected to immunoblotting using the indicated antibodies 48 h later. Data are presented as the mean ± SD and analyzed using one-way analysis of variance with the Bonferroni post-hoc test. $^{*}P < 0.05$, $^{**}P < 0.01$, three independent experiments. The levels of lipidated LC3 (LC3 II) were quantitated after normalization with the control as 1.00. TRIM72 tripartite motif-containing 72

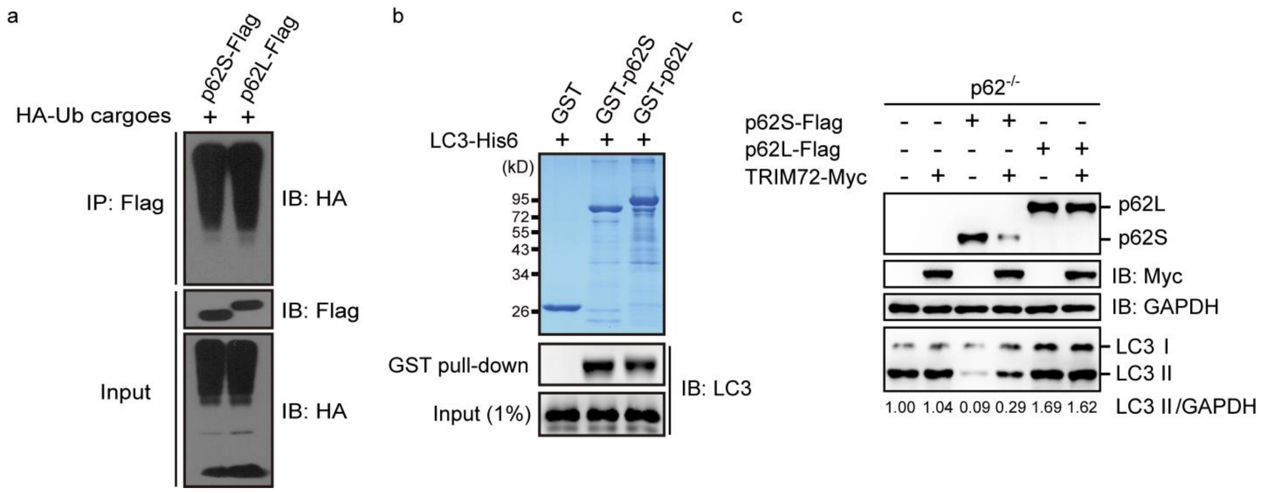

Fig. S4 Human p62S antagonizes the autophagy receptor function of p62L. **a** Human p62S bound to and pulled down poly-ubiquitinated cargo as efficiently as p62L. HeLa cells expressing HA-tagged ubiquitin (Ub) conjugates were affinity-enriched with anti-HA chromatography. Flag-tagged p62S or p62L were incubated with the HA-tagged Ub conjugates, immunoprecipitated with anti-Flag affinity gels, followed by immunoblotting analysis using the indicated antibodies. **b** Human p62S was capable of binding with LC3, despite lacking the PB1 domain at its N-terminus. Glutathione-S-transferase (GST) pull-down assays were conducted using recombinant GST-tagged p62S or p62L and His6-tagged LC3. GST was a negative control. **c** Human p62S was required for autophagy activated by TRIM72. The $p62^{-/-}$ MEF cells were transiently co-transfected with TRIM72 and p62L or p62S, and subjected to immunoblotting using the indicated antibodies 48 h later. The levels of lipidated LC3 were quantitated after normalization of the control as 1.00. TRIM72 tripartite motif-containing 72

Table S1 Sequences of the primers used in RT-PCR and qPCR

Target gene	Forward (5'-3')	Reverse (5'-3')
GAPDH	GAGTCAACGGATTTGGTCGTATTG	ATTTGCCATGGGTGGAATCATATTG
p62L	GCCTACCTTCTGGGCAAGGA	ACTGGAAAAGGCAACCAAGTC
p62S	CTGAACTAAGGAGAAAGTCCTACA	GTTCCTACCACAGGCCCATT

RT-PCR reverse transcription PCR, *qPCR* quantitative PCR

Table S2 Sequences of the shRNAs for p62S and TRIM72

shRNA	Target site sequence (5'-3')
Scramble	GCGCGATAGCGCTAATAATTT
shp62S	CAGTTTGGCGTGCAACATGGG
shTRIM72	CAGACTGAGTTCCTCATGAAA

TRIM72 tripartite motif-containing 72
